# Linking aberrant glycosylation of plasma glycoproteins with progression of myelodysplastic syndromes: a study based on plasmonic biosensor and lectin array

**DOI:** 10.1038/s41598-023-39927-4

**Published:** 2023-08-07

**Authors:** Leona Chrastinová, Ondřej Pastva, Markéta Bocková, Hana Kovářová, Eliška Ceznerová, Roman Kotlín, Pavla Pecherková, Jana Štikarová, Alžběta Hlaváčková, Marek Havlíček, Jan Válka, Jiří Homola, Jiří Suttnar

**Affiliations:** 1 Institute of Hematology and Blood Transfusion, Prague, Czech Republic; 2 Institute of Photonics and Electronics, Czech Academy of Sciences, Prague, Czech Republic; 3 Department of Biochemistry, Institute of Hematology and Blood Transfusion, U Nemocnice 1, 128 20 Prague 2, Czech Republic

**Keywords:** Biomarkers, Oncology

## Abstract

Aberrant glycosylation of glycoproteins has been linked with various pathologies. Therefore, understanding the relationship between aberrant glycosylation patterns and the onset and progression of the disease is an important research goal that may provide insights into cancer diagnosis and new therapy development. In this study, we use a surface plasmon resonance imaging biosensor and a lectin array to investigate aberrant glycosylation patterns associated with oncohematological disease—myelodysplastic syndromes (MDS). In particular, we detected the interaction between the lectins and glycoproteins present in the blood plasma of patients (three MDS subgroups with different risks of progression to acute myeloid leukemia (AML) and AML patients) and healthy controls. The interaction with lectins from *Aleuria aurantia* (AAL) and *Erythrina cristagalli* was more pronounced for plasma samples of the MDS and AML patients, and there was a significant difference between the sensor response to the interaction of AAL with blood plasma from low and medium-risk MDS patients and healthy controls. Our data also suggest that progression from MDS to AML is accompanied by sialylation of glycoproteins and increased levels of truncated O-glycans and that the number of lectins that allow discriminating different stages of disease increases as the disease progresses.

## Introduction

Glycosylation is one of the most common post-translational modifications, along with phosphorylation and acetylation. It is estimated that 70–80% of all human proteins and > 85% of secretory proteins are glycosylated^1^. Glycosylation is designated as N-linked or O-linked depending on whether the glycosidic moiety occurs at asparagine amino acid residues (N-glycans) or serine/threonine residues (O-glycans). Enzymatic protein glycosylation occurs in the endoplasmic reticulum and the Golgi complex, and is affected by the expression and activity of glycosidases and glycosyltransferases^2^. Glycans participate in various physiological and pathological processes, such as intermolecular and cell–cell recognition events. Glycans have also been shown to affect the cell cycle, differentiation and apoptosis, host–pathogen interactions and inflammation^3,4^. In addition, it has been hypothesised that glycans can indicate the fitness of a maturing protein^5^. Glycans also play a role in the inactivation of proteins present in the blood; for example, fibrinogen has been shown to aggregate and become biologically inactive following de-N-glycosylation^6,7^.

Glycan structure or glycosidic moiety composition (referred to here as glycan aberration) is an important topic for research since there is a correlation between specific glycan aberrations and morbidity and life expectancy in patients with cancer^8^. Typical cancer-related glycan aberrations include truncated O-glycans such as T, sialyl Tn (STn), or Tn antigens, as well as N-glycans modified with core fucose, and bisecting and branching N-glycans^9^. In addition, glycan chains may be capped with terminal glycan epitopes such as fucosylated Lewis (Le) and sialyl Lewis (SLe) antigens^10,11^. Glycan aberrations may influence the biological functions of both plasma proteins (e.g., immunoglobulin G)^12,13^ and cell adhesion proteins (e.g., integrins or cadherins involved in cancer cell invasion)^14^. Specific glycan aberrations have also been involved in the oncogenic activation of receptor tyrosine kinases and the regulation of key processes in malignant transformation and cancer progression^15,16^.

A crucial factor in the development of haematological malignancies is the interaction between the malignant cells and the bone marrow. Aberrant glycan features, such as hypersialylation and hyperfucosylation, are often associated with the interaction of neoplastic cells with the microenvironment^17^. Aberrant glycan expression or altered transcription of glycosyltransferase (GT) and glycosidase genes were found in blood plasma and/or serum of patients with chronic lymphocytic leukaemia (CLL, N-acetylgalactosaminyltransferase 11)^18^, chronic myeloid leukaemia (CML, N-acetylglukosaminyltransferase-III)^19^, acute lymphocytic leukaemia (sialyltransferases)^20^, and AML (sialyltransferases)^21^. Myelodysplastic syndromes are a heterogeneous group of haematological malignancies affecting pluripotent haematopoietic stem cells with a high risk of progression to AML^22^. MDS is associated with mutations^23^, changes in protein concentrations, and post-translational protein modification^24,25^.

A number of methods have been used to investigate glycan structure, including nuclear magnetic resonance, mass spectrometry, chromatography, and electrophoresis. However, due to the large structural variability of glycans and similarities in the physicochemical properties of glycan building blocks, the determination of glycan structure is challenging and time-consuming^26^. Recently, methods based on lectins, a diverse family of proteins, have attracted a great deal of attention. Lectins can recognise various types of free glycans as well as glycans attached to glycoconjugates and can distinguish monosaccharides (e.g., sialic acid, fucose, mannose, N-acetylglucosamine) as well as structural moieties (monosaccharide linkages, branching) within the glycan chain^1,27^. In 2005, Kuno et al. reported using a lectin microarray and fluorescence readout method for glycan profiling^28^. Subsequently, lectin microarrays were combined with a variety of readout methods, including electrochemical impedance spectroscopy^29,30^, quartz crystal microbalance^31^, and surface plasmon resonance (SPR)^32,33^. SPR is an optical method that allows real-time and label-free monitoring of biomolecular interactions and quantification of biomolecules^33^. The SPR sensor platform that is most compatible with that array format is SPR imaging (SPRi), which allows parallel investigation of biomolecules and their interactions in a temporally and spatially resolved manner^34,35^. SPR sensor methods have been combined with lectins to study lectin binding to specific glycan aberrations and the effect of lectins on the proliferation of leukaemic cells^36^.

In this work, we combine a lectin array and SPRi biosensor platform to investigate the interactions between selected lectins and blood plasma of oncohematological patients (three MDS subgroups and MDS progressed into AML) and healthy donors to evaluate the link between aberrant glycosylation of plasma glycoproteins and MDS progression.

## Results and discussion

**Figure 1:**
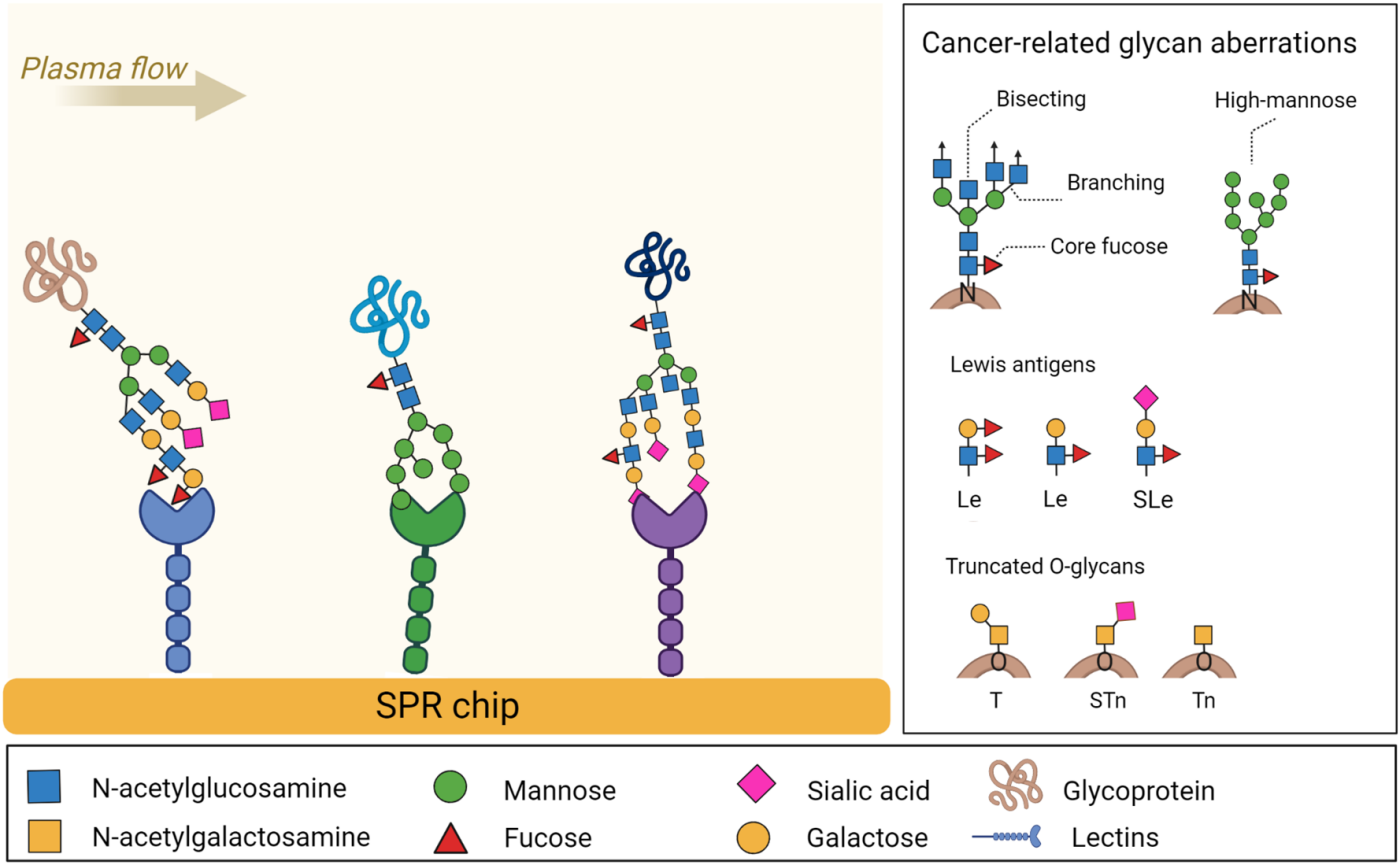
The principle of lectin array for the detection of glycan aberrations in blood plasma. Selected lectins show affinity to cancer glycan aberrations (inset), such as bisecting, increased branching, high mannose and core fucose of N-glycans, the capping of glycan chains with terminal glycan epitopes, such as fucosylated Lewis (Le) antigens and sialyl Lewis (SLe) antigens, and truncated O-glycans such as T, sialyl Tn (STn), and Tn antigens.

Figure 1 shows a schematic diagram of the detection of cancer-related glycan aberrations using a lectin chip. A lectin array (for details, see Table 1) on the surface of an SPR chip is exposed to blood plasma. Glycans with different aberrations bind to distinct lectins giving rise to a sensor response in corresponding parts of the array.

**Table 1:** Lectins used in the assay.

Lectin from	Symbol	Affinity for	Aberration of glycan
*Erythrina cristagalli*	ECL	Gal, Gal-GlcNAc	Galactosylation
*Triticum vulgaris*	WGA	GlcNAc	Branching
*Maackia amurensis*	MAL	NeuAc	Sialylation
*Lens culinaris*	LCH	Man, Glc	High man, core fuc
*Aleuria aurantia*	AAL	Fuc-GlcNAc, Fuc-LacNAc	Core fuc, Lewis antigen
*Ulex europaeus*	UEA	Fuc	Lewis antigen
*Arachis hypogaea*	PNA	Gal-GalNAc	T, ST, Tn antigen
*Bandeiraea simplicifolia*	BSL	Gal, GalNAc	Tn, STn antigen
*Viccia villosa*	VVL	GalNAc	Tn antigen
*Helix pomatia*	HPA	GalNAc	T, Tn antigen

Fucose (Fuc), galactose (Gal), glucose (Glc), mannose (Man), lactose (Lac), N‐acetylneuraminic acid (NeuAc), N-acetyl (NAc), sialyl group (S).

**Figure 2:**
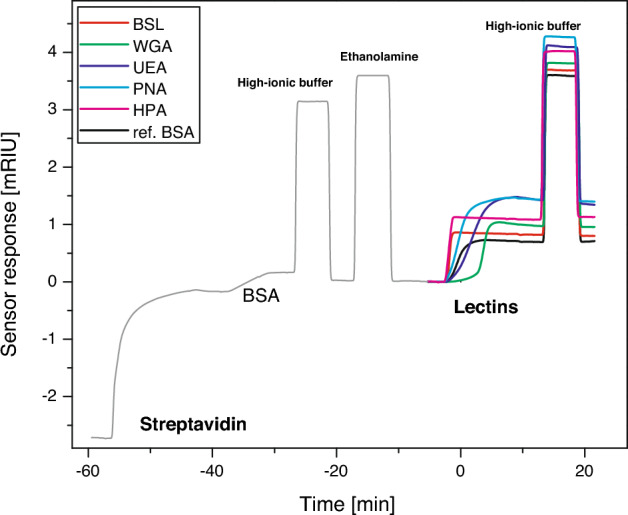
Sensorgram corresponding to the immobilisation of different lectins (BSL, WGA, UEA, PNA, HPA). The sensor surface in the reference channel was coated with BSA.

The sensorgram in Fig. 2 shows the sensor response to the immobilisation of lectins in five sensing channels, with immobilisation of bovine serum albumin (BSA) shown as a reference. The final level of the immobilised proteins was determined from the sensor response in phosphate-buffered saline (PBS) 5 min after the sensor surface was treated with a high ionic strength buffer. The quantity of each lectin immobilised on different chips was reproducible; the standard deviation calculated using data from five different chips was 12% or less.

We then measured interactions between the immobilised lectins and blood plasma. The plasma samples were obtained from four groups of patients with MDS and AML (divided according to the 2016 WHO classification) and one group of healthy donors. The four patient groups were as follows: (1) MDS with refractory anaemia and refractory anaemia with ringed sideroblasts (MDS-SLD, low-risk of MDS progression); (2) refractory cytopenia with multilineage dysplasia (MDS-MLD, medium-risk of MDS progression); (3) refractory anaemia with excess blasts (MDS-EB, high-risk of MDS progression); and (4) MDS patients who had progressed to AML (Table 2, see “Methods”).

**Table 2:** Demographic and clinical characteristics of patients and healthy donors.

Probands	All	Male	Female	p-value
	(N = 70)	(N = 46)	(N = 24)	
Age (median, min–max)	70 (30–87)	71.5 (30–87)	62 (30–80)	0.018*
Diagnosis—N (%)				
Control	13 (18.6)	8 (17.4)	5 (20.8)	NS**
MDS-SLD	5 (7.1)	2 (4.4)	3 (12.5)	
MDS-MLD	17 (24.3)	12 (26.1)	5 (20.8)	
MDS-EB	19 (27.1)	14 (30.4)	5 (20.8)	
AML	16 (22.9)	10 (21.7)	6 (25.0)	

*Mann–Whitney test, **Chi-square test, NS—non-significant, MDS-SLD—MDS with single lineage dysplasia, MDS-MLD—MDS with multilineage dysplasia, MDS-EB—MDS with excess blasts, included three patients with MDS-EB-1 and sixteen with MDS-EB-2. The MDS-SLD group consisted of two patients with refractory anaemia and three patients with refractory anaemia with ring sideroblasts. AML patients have been documented to progress from MDS to AML.

**Figure 3:**
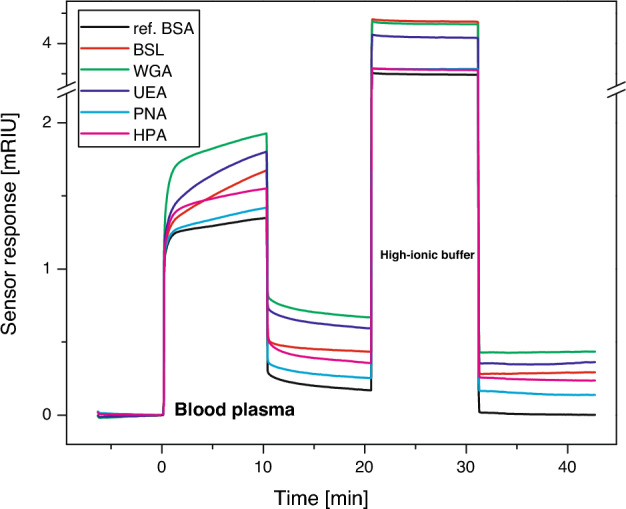
Sensor responses to a blood plasma sample (MDS-EB) interacting with five different lectins. The sensor surface in the reference channel was coated with BSA.

The sensorgram in Fig. 3 shows the sensor response to interactions between the five different immobilised lectins and a plasma sample. The final level of interacting plasma proteins was determined from the sensor response in PBS 10 min after the sensor surface was treated with a high ionic strength buffer. Typical responses were rather strong and larger than the baseline noise of the SPR sensor by a factor of ≥ 130. We determined the chip-to-chip reproducibility of the sensor response to be around 84% for all lectins (calculated using data from three different chips). To process the data, we first normalised the sensor responses to the plasma samples according to the respective levels of immobilised lectins. Then, to suppress the effects of non-specific interactions and interference from other factors, such as bulk refractive index variations and temperature fluctuations, we subtracted the response in the reference channel (with immobilised BSA) from that in the sensing channel (with the immobilised lectin) yielding the reference-compensated sensor response (SR).

**Figure 4:**
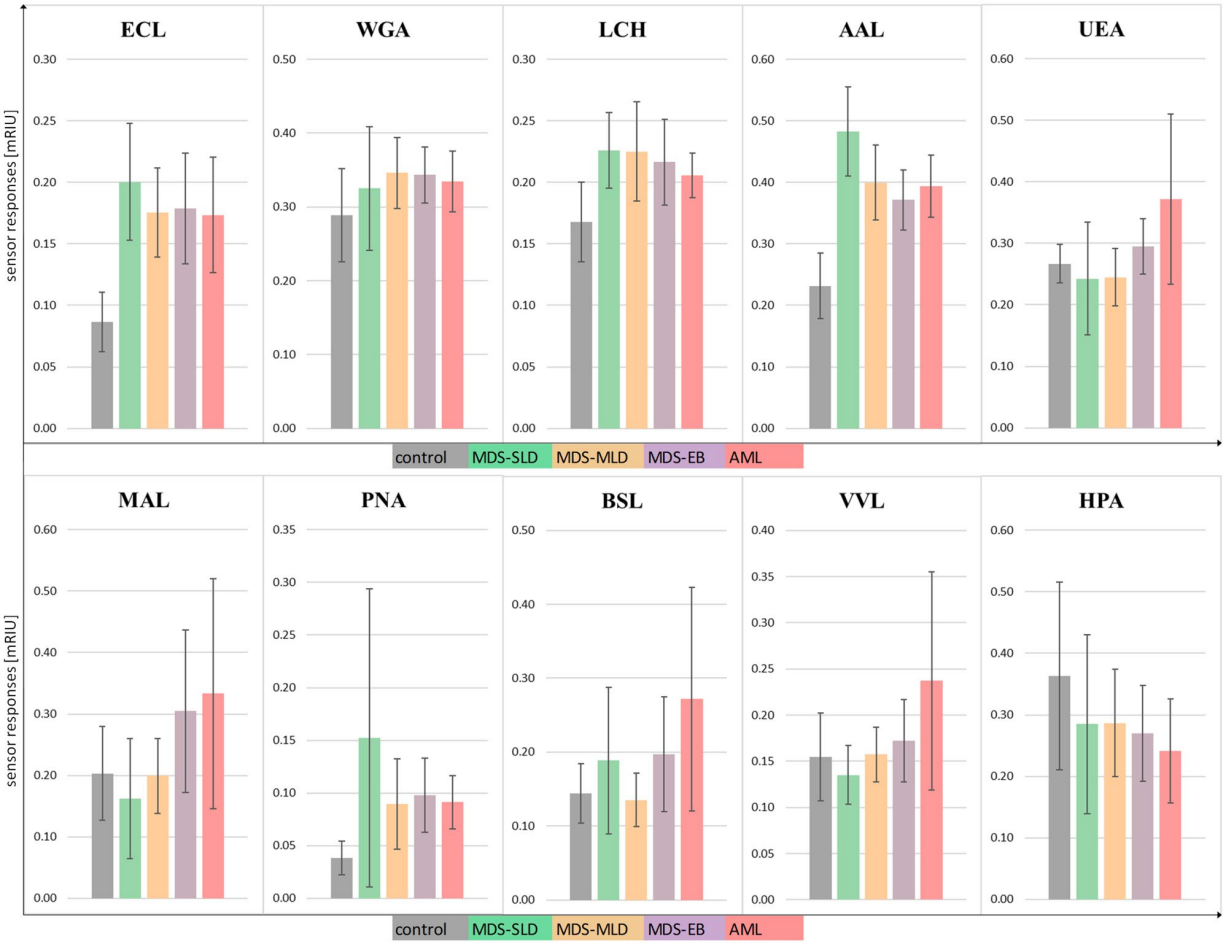
Sensor responses (mRIU) to blood plasma interacting with immobilised lectins. Ten different lectins were used: ECL, WGA, LCH, AAL, UEA, MAL, PNA, BSL, VVL and HPA. Blood samples from healthy donors and from patients with MDS-SLD (low-risk), MDS-MLD (medium-risk) and MDS-EB (high-risk), and patients who had already progressed from MDS to AML were tested. Data are presented as mean values plus 90% confidence interval.

Figure 4 shows SRs for each lectin to plasma samples from the five different groups. The graphs show mean values plus a 90% confidence interval for all tested groups (Table S1 in the Supplementary Information). We found increased SRs to lectins from *Erythrina cristagalli* (ECL), *Triticum vulgaris* (WGA), *Lens culinaris* (LCH), AAL, and *Arachis hypogaea* (PNA), and decreased SRs to lectins from *Helix pomatia* (HPA) for all patient groups when compared to healthy donors. In addition, SRs to *Ulex europaeus* (UEA), *Bandeiraea simplicifolia* (BSL) and *Vicia villosa* (VVL) were increased for the AML group compared with the MDS and healthy donor groups. Furthermore, SRs to *Maackia amurensis* (MAL) were greater for the MDS-EB and AML groups compared with the MDS-SLD, MDS-MLD and healthy donor groups.

**Figure 5:**
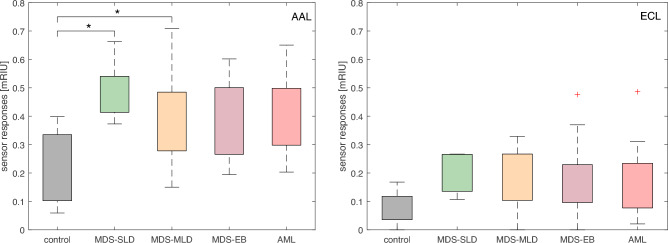
Box plots of sensor responses to blood plasma interacting with immobilised lectins. SRs (mRIU) to plasma samples from MDS-SLD (low-risk), MDS-MLD (medium-risk), MDS-EB (high-risk), AML and healthy donor groups with immobilised lectins AAL (Left) and ECL (Right). Data are presented as medians with minimum and maximum values. *P < 0.05.

To perform statistical analysis of SRs obtained across the plasma samples from the five groups, we first applied the Shapiro normality test to the SRs data obtained from all clinical samples for all lectins. This showed that the SRs were not normally distributed and we therefore analysed the data using the Kruskal–Wallis test. The analysis showed significant differences in SRs among the groups for lectins AAL and ECL (Kruskal–Wallis test, P = 0.009, P = 0.050, respectively). Then, using a *post-hoc* test, we found that SRs to immobilised AAL were significantly higher in the MDS-SLD and MDS-MLD groups when compared with the healthy donor group (Tukey–Kramer test, P = 0.015, P = 0.041, respectively) (Fig. 5).

Our experiments showed significant differences among the MDS groups in SRs to two lectins, AAL and ECL. For AAL, levels of interacting proteins in plasma from the MDS-SLD and MDS-MLD groups were significantly greater than levels from healthy donors. AAL recognises fucosylation of glycans (core fucosylation and terminal fucosylation—Le antigen) and ECL exhibits high affinity for Gal-GlcNAc, which is typical of galactosylated N-glycans^37^. Consequently, these results suggest that fucosylation of glycoproteins and galactosylated N-glycans are the most pronounced aberrations in the plasma of MDS patients. These findings correspond well with the results of previous studies reported in the literature. For example, Pang et al. proposed that the three processes most commonly involved in aberrant glycosylation in haematological malignancies are fucosylation, sialylation and bisecting GlcNAc^17^. Core fucosylation of N-glycans is carried out by fucosyltransferase 8 (FUT8). FUT8 also inhibits haemoglobin production and erythroid differentiation of leukaemia cells during haematopoiesis^38^, which is a characteristic of MDS-SLD^39^. N-glycan structures have a unique feature, a large number of potential branches. GlcNAc branches are usually elongated by galactose and sialic acid, whereas bisecting GlcNAc is not futher elongated. Aberrations of these branches are often found in cancer cells^40^. Polylactosamine structure [Gal-GlcNAc]n act as a high-affinity ligand for galectins which are important for growth factor signaling and cell proliferation^41^.

Based on the data collected in Fig. 4, the development and progression of MDS are likely to be accompanied by other glycan modifications in addition to terminal and core fucosylation and galactosylated N-glycans. We observed increased SRs for all patient groups in comparison with healthy donors for AAL (core and terminal fucose), LCH (core fucose, mannose), ECL (galactosylated N-glycans), WGA (branching^42^) and PNA (T, ST and Tn antigen). SRs in the AML group were different from the MDS groups and healthy donors for UEA (terminal fucose), BSL (Tn antigen and STn antigen), and VVL (Tn antigen). SRs were increased for MAL (sialic acid) in the MDS-EB and AML groups compared with the other groups. Interestingly, decreased SRs were observed for all patient groups (when compared to healthy donors) for HPA (Tn antigen). Because SRs were increased for PNA in all MDS subtypes and AML, for MAL in MDS-EB and AML, and for BSL in AML and because these lectins have an affinity for glycans containing sialic acid, we suppose that sialylation levels increase during MDS progression. Moreover, the progression of MDS to AML is probably accompanied by increased levels of truncated O-glycans (PNA, BSL, VVL—T, ST, Tn and STn antigen). Several studies have examined the level of sialylation in blood and tissue samples from patients with different cancers. Patel et al. observed an elevated sialic acid/total protein ratio, as well as elevations in lipid-bound sialic acid and fucose/total protein ratio in patients with anaemia (moderate elevations) and patients with untreated leukaemia (substantial elevations) when compared with controls^43^. It was also reported that levels of sialyltransferases are elevated in mononuclear cells of patients with CML (these cells are known to be associated with resistance to the tyrosine kinase inhibitor imatinib)^44^. Barbier et al. examined AML cell lines and concluded that the interaction between SLe antigen and E-selectin is the main cause of chemoresistance in AML^45^. In addition, a study of N-glycans sialylation in adriamycin-resistant AML cell lines demonstrated a role for sialylation in chemoresistance^46^. Several studies have examined levels of truncated O-glycans (T, ST, Tn, and STn antigen) in various cancers. Aller et al. reported that Tn antigen is strongly expressed in CLL^47^, and elevated levels of T, Tn, and STn antigens have been observed in both tissue and serum samples from patients with gastric cancers^48^. In addition, Simplicien et al. found that HPA lectin (T/Tn-specific) kills Tn-positive leukaemia cells and Tn-negative lymphoma cells, but does not affect healthy lymphocytes^49^. Our results show increased SRs to PNA (T, ST and Tn antigen) and BSL (Tn and STn antigen), which we believe to be caused by increased sialylation and the presence of ST and STn antigens in plasma. Our study revealed a substantial difference in the glycosylation patterns of the MDS/AML patients and healthy controls. We also found a difference in the glycosylation patterns between MDS-SLD/MLD (low/medium-risk) group and the MDS-EB (high-risk) group whose glycosylation pattern is more similar to that of the AML group.

**Figure 6:**
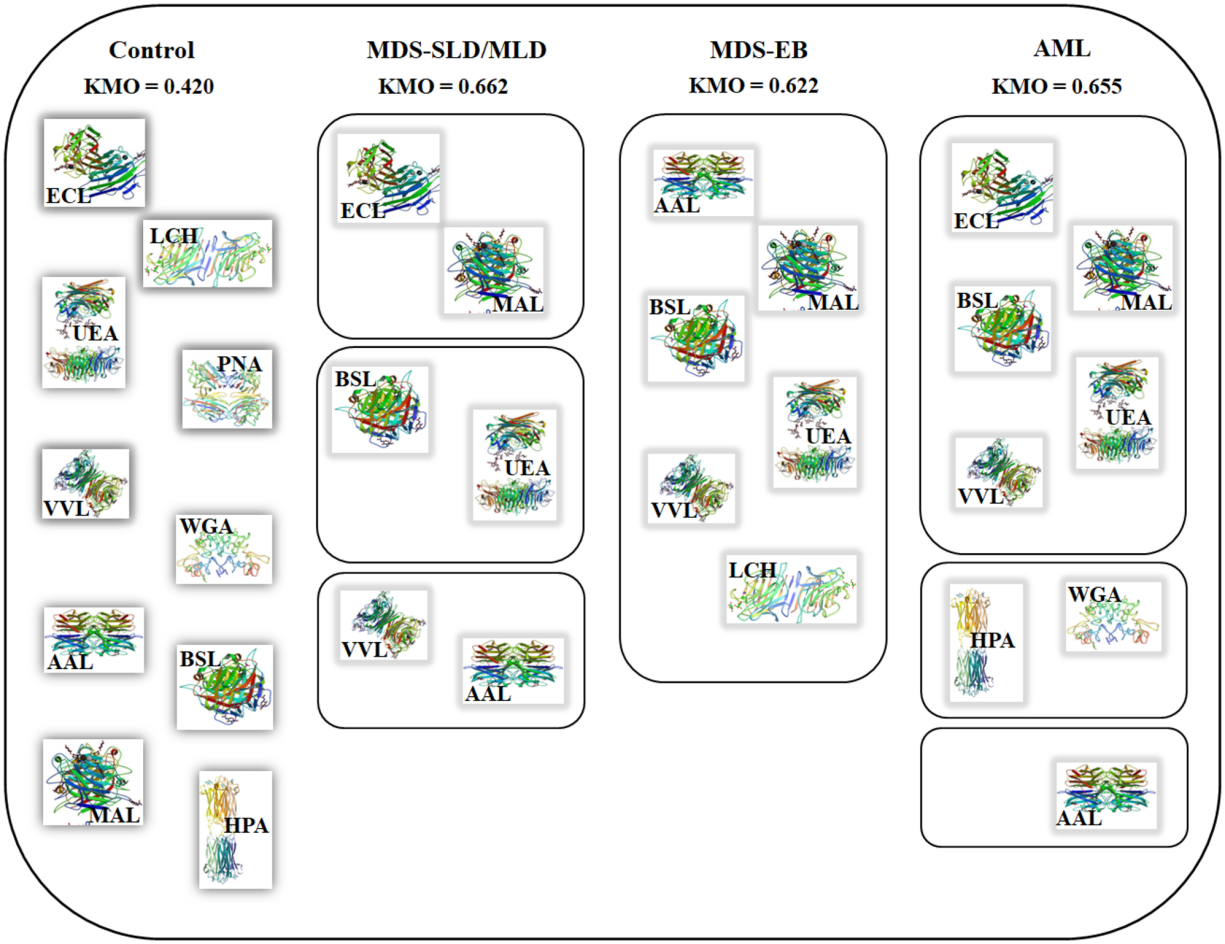
Graphical representation of the results of factor analysis. Lectins placed in the boxes are specific to the diagnosis group and their SRs correlate among each other. Lectins are represented by symbols and 3D structures. Tested groups—healthy donors, patients with MDS-SLD/MLD (low and medium-risk), patients with MDS-EB (high-risk), and patients who had already progressed to AML.

We used factor analysis to evaluate the relationship between trends for SRs to individual lectins observed in the different stages of MDS. In the first step of the analysis, we calculated correlations between SRs to each combination of lectins for each group of plasma samples. The MDS-SLD and MDS-MLD groups were combined as there were not enough patients for factor analysis in the MDS-SLD group (see Tables S2–S5 in the Supplementary Information for results). A Kaiser–Meyer–Olkin (KMO) data suitability test was performed, and only groups with a KMO value > 0.5 were included in the factor analysis (this condition was fulfilled for all patient groups but not for the healthy donors). In the second step of the factor analysis, groups of lectins characteristic for each stage of the disease were identified based on the features in SRs trends. The results are illustrated in Fig. 6. The MDS-SLD/MLD (low/medium-risk) group was characterised by six lectins: ECL, MAL, AAL, VVL, BSL and UEA. These lectins belonged to three different components of the factor analysis, which means that the correlation between SRs was apparent for three different pairs, specifically, ECL and MAL, AAL and VVL, and BSL and UEA. Six lectins were found to be specific for the MDS-EB (high-risk) group: MAL, AAL, VVL, BSL, UEA and LCH. These lectins belonged to a single component of the factor analysis, i.e., SRs for all these six lectins were found to be correlated among each other. For patients who had already progressed to AML, we identified eight specific lectins: ECL, MAL, VVL, BSL, UEA, HPA, WGA and AAL. Five of the eight lectins belonged to one component of the factor analysis and their SRs were correlated with each other (ECL, MAL, VVL, BSL and UEA); two lectins belonged to another component and were correlated among themselves (HPA and WGA). These findings indicate that the number of correlated SRs pertinent to group characteristic lectins increases with the MDS disease severity. Therefore, the signature of glycan aberrations in plasma could provide some diagnostic benefit for MDS.

## Conclusions

To advance understanding of the relationship between aberrations of glycosylation and MDS progression, we investigated changes in glycosylation patterns in the blood plasma of patients with MDS and AML using an SPR imaging biosensor and lectin array. Our study showed significantly increased levels of fucosylation and galactosylation of N-glycans in patients with MDS and AML. In addition, we used factor analysis to identify lectins that may be important biomolecular reporters for different stages of MSD and found that interactions between selected lectins and glycoproteins in blood plasma of patient subgroups (MDS-SLD/MLD, MDS-EB and AML) were correlated. This work demonstrates the potential of lectin-based technology for the investigation of glycan aberrations and research into molecular processes associated with MDS.

## Methods

### Chemicals and reagents

The lectins UEA, WGA, PNA, HPA, BSL and streptavidin from *Streptomyces avidinii* were purchased from Sigma-Aldrich (Prague, Czech Republic). Lectins AAL, ECL, LCH, MAL and VVL were purchased from GALAB Technologies GmbH (Hamburg, Germany) (Table 1), prepared aliquots of biotinylated lectins were stored at − 20 °C.

The 16-Mercapto-hexa(ethylene glycol) hexadecanoic acid (HSC_11_(EG)_6_OCH_2_COOH) and 11-mercapto-tetra(ethylene glycol)undecanol (HSC_11_(EG)_4_OH) were purchased from Prochimia (Gdansk, Poland). Bovine serum albumin (BSA) was purchased from Sigma-Aldrich (Prague, Czech Republic). Ethanolamine hydrochloride (EA), 1-hydroxypyrrolidine-2,5-dione (NHS) and 3-(ethyliminomethylideneamino)-N,N-dimethylpropan-1-amine (EDC) were purchased from Cytiva (Uppsala, Sweden).

### Blood plasma samples

Blood samples were collected from patients with MDS diagnosed at the Institute of Hematology and Blood Transfusion, Prague, Czech Republic, and from healthy donors. Informed consent was obtained from all study participants at the time of blood collection. The study was approved by the Institute of Hematology and Blood Transfusion Ethics Committee, and all samples were obtained in accordance with the regulations of the ethics committee of the institute and with the Declaration of Helsinki.

Human blood was drawn from patients and healthy donors by venipuncture into polypropylene tubes coated with EDTA. Plasma was obtained by centrifugation (5 min, 2880×*g*) and was stored at − 70 °C until analysis. Details of the patients and donors providing the plasma samples used in this study are in Table 2.

### SPR biosensor platforms

SPR biosensor platform developed at the Institute of Photonics and Electronics (Prague, Czech Republic) was used in this study. A high-resolution SPR imaging system with polarisation contrast and internal referencing^50^ was used to investigate protein–protein interactions in MDS plasma samples. This SPRi system enables simultaneous analysis of biomolecular interactions in 6 × 6 individual flow-through sensing spots, using two flow cells. One flow cell was used for surface functionalisation, while the second was used for MDS plasma analysis. The response of the SPRi biosensor is expressed in refractive index units (RIU), which are related to the surface concentration of biomolecules on the sensor surface^50^. The SPRi biosensor used in these experiments exhibited noise with a standard deviation of 6 × 10^–7^ RIU (calculated from the sensor response baseline).

The SPR sensor was equipped with a temperature controller, and sample delivery to the sensor was carried out using a microfluidic flow cell in a near-dispersionless manner^51^. The flow rate was 20 µl/min unless otherwise stated. We employed a reference channel in each experiment to compensate for interfering effects. In the reference channel, the surface was functionalised with covalently attached biotinylated BSA alone.

### Immobilisation of lectins on the SPR chip

In all experiments, lectins were immobilised via covalent attachment to a ω-carboxyalkylthiolate self-assembled monolayer (SAM). Details of the mixed SAM preparation of HS-C11-(EG)_4_-OH and HS-C11-(EG)_6_-OCH_2_-COOH alkylthiols, as well as the in situ activation of carboxylic terminal groups, have been described previously^52,53^. After activation, the chip surface was incubated with SA_5_ and streptavidin (50 μg/ml). BSA was covalently attached (5 μg/ml BSA in SA_5_ for 5 min) to increase the surface resistance to non-specific adsorption. High ionic strength PBNa was injected for 5 min to remove non-covalently bound streptavidin and BSA. Finally, the sensor surface was treated with 1 M EA for 5 min to deactivate the remaining carboxylic groups. Immobilisation of the biotinylated lectins was performed in PBS for 12 min (flow rate 5 µl/min, lectin concentration 5 μg/ml). High ionic strength PBNa was then injected for 5 min to remove loosely bound lectins.

### Detection of interacting glycoproteins

After the immobilisation of lectins, we investigated their interaction with glycoproteins in blood plasma samples. Before the plasma sample was injected, we flowed PBS_BSA_ across the sensor surface until a stable baseline was reached. Plasma (diluted tenfold with PBS_BSA_ to achieve the best ratio between specific and non-specific sensor responses) was then injected for 10 min, followed by injection of PBS_BSA_. Finally, we flowed high ionic strength PBNa over the sensor for 10 min, followed by the PBS_BSA_ running buffer.

### Statistical analysis

The statistical analysis of SR data obtained from all clinical samples across the lectins was performed using Shapiro normality test, non-parametric Mann–Whitney and Kruskal–Wallis tests, followed by a *post-hoc* Tukey–Kramer multiple comparison test. Gender independence was assessed with a Chi-square test.

Several statistical approaches were combined to ensure a complete analysis of the data, including factor analysis, correlation analysis and hypothesis testing. Data reduction using factorial analysis was used to identify a small number of factors (latent variables) that could explain most of the variance observed in a much larger number of manifest variables. To use factor analysis, the data had to meet four basic assumptions: (i) several variables were continuous or at least ordinal, (ii) there was a linear relationship between the variables (this was tested using a matrix scatterplot), (iii) the appropriate number was additionally tested using the Kaiser–Meyer–Olkin Sampling Adequacy, (iv) some variables were correlated with each other (this was verified using the correlation matrix and also verified by Bartlett's test of sphericity). We verified that there were no outliers in the data that could affect the results by checking standard deviations. Only factors with eigenvalues > 1 were used for further analysis. No rotation method was used. Factor loading was set to an absolute value of 0.5.

The statistical methods were performed using IBM SPSS Statistics for Windows, Version 23.0. (IBM, Armonk, NY) and graphs were drawn using MATLAB (2021) 9.10.0.1684407, R2021a (The MathWorks, Inc, Natick, Massachusetts). All statistical significance tests were standardized at an alpha level of P < 0.05.

### Supplementary Information

Supplementary Tables.

## Data Availability

The data that support the findings of the current study are available from the corresponding author upon request.

## References

[1] Belický Š, Katrlík J, Tkáč J (2016). Glycan and lectin biosensors. Essays Biochem..

[2] Schjoldager KT, Narimatsu Y, Joshi HJ, Clausen H (2020). Global view of human protein glycosylation pathways and functions. Nat. Rev. Mol. Cell Biol..

[3] Maverakis E (2015). Glycans in the immune system and The Altered Glycan Theory of Autoimmunity: A critical review. J. Autoimmun..

[4] Taniguchi N, Kizuka Y (2015). Glycans and cancer: Role of N-Glycans in cancer biomarker, progression and metastasis, and therapeutics. Adv. Cancer Res..

[5] Hebert DN, Molinari M (2012). Flagging and docking: Dual roles for N-glycans in protein quality control and cellular proteostasis. Trends Biochem. Sci..

[6] Berger M, Kaup M, Blanchard V (2012). Protein glycosylation and its impact on biotechnology. Adv. Biochem. Eng. Biotechnol..

[7] Jayaprakash NG, Surolia A (2017). Role of glycosylation in nucleating protein folding and stability. Biochem. J..

[8] Freeze HH, Kinoshita T, Varki A, Varki A (2009). Glycans in acquired human diseases. Essentials of Glycobiology, Chapter 43.

[9] Mereiter S, Balmaña M, Campos D, Gomes J, Reis CA (2019). Glycosylation in the era of cancer-targeted therapy: Where are we heading?. Cancer Cell.

[10] Zhang Z, Wuhrer M, Holst S (2018). Serum sialylation changes in cancer. Glycoconj. J..

[11] Yogeeswaran G, Salk PL (1981). Metastatic potential is positively correlated with cell surface sialylation of cultured murine tumor cell lines. Science.

[12] Kaneko Y, Nimmerjahn F, Ravetch JV (2006). Anti-inflammatory activity of immunoglobulin G resulting from Fc sialylation. Science.

[13] Shields RL (2002). Lack of fucose on human IgG1 N-linked oligosaccharide improves binding to human FcγRIII and antibody-dependent cellular toxicity. J. Biol. Chem..

[14] Carvalho S, Reis CA, Pinho SS (2016). Cadherins glycans in cancer: Sweet players in a bitter process. Trends Cancer.

[15] Mereiter S (2016). Glycomic analysis of gastric carcinoma cells discloses glycans as modulators of RON receptor tyrosine kinase activation in cancer. Biochim. Biophys. Acta Gen. Subj..

[16] Rodrigues JG (2018). Glycosylation in cancer: Selected roles in tumour progression, immune modulation and metastasis. Cell. Immunol..

[17] Pang X, Li H, Guan F, Li X (2018). Multiple roles of glycans in hematological malignancies. Front. Oncol..

[18] Libisch MG (2014). GALNT11 as a new molecular marker in chronic lymphocytic leukemia. Gene.

[19] Yoshimura M (1995). High expression of UDP-N-acetylglucosamine: Beta-d mannoside beta-1,4-N-acetylglucosaminyltransferase III (GnT-III) in chronic myelogenous leukemia in blast crisis. Int. J. Cancer.

[20] Mondal S, Chandra S, Mandal C (2010). Elevated mRNA level of hST6Gal I and hST3Gal V positively correlates with the high risk of pediatric acute leukemia. Leuk. Res..

[21] Wang D (2022). Glycosphingolipid-glycan signatures of acute myeloid leukemia cell lines reflect hematopoietic differentiation. J. Proteome Res..

[22] Tefferi A, Vardiman JW (2009). Myelodysplastic syndromes. N. Engl. J. Med..

[23] Ogawa S (2019). Genetics of MDS. Blood.

[24] Hlaváčková A (2017). Enhanced plasma protein carbonylation in patients with myelodysplastic syndromes. Free Radic. Biol. Med..

[25] Aasebø E, Forthun RB, Berven F, Selheim F, Hernandez-Valladares M (2016). Global cell proteome profiling, phospho-signaling and quantitative proteomics for identification of new biomarkers in acute myeloid leukemia patients. Curr. Pharm. Biotechnol..

[26] Alley WRJ, Mann BF, Novotny MV (2013). High-sensitivity analytical approaches for the structural characterization of glycoproteins. Chem. Rev..

[27] André S, Kaltner H, Manning JC, Murphy PV, Gabius H-J (2015). Lectins: Getting familiar with translators of the sugar code. Molecules.

[28] Kuno A (2005). Evanescent-field fluorescence-assisted lectin microarray: A new strategy for glycan profiling. Nat. Methods.

[29] Paleček E (2015). Electrochemistry of nonconjugated proteins and glycoproteins. Toward sensors for biomedicine and glycomics. Chem. Rev..

[30] Pihíková D, Kasák P, Tkac J (2015). Glycoprofiling of cancer biomarkers: Label-free electrochemical lectin-based biosensors. Open Chem..

[31] Mi F (2021). Application of lectin-based biosensor technology in the detection of foodborne pathogenic bacteria: A review. Analyst.

[32] Safina G (2012). Application of surface plasmon resonance for the detection of carbohydrates, glycoconjugates, and measurement of the carbohydrate-specific interactions: A comparison with conventional analytical techniques. A critical review. Anal. Chim. Acta.

[33] Homola J (2008). Surface plasmon resonance sensors for detection of chemical and biological species. Chem. Rev..

[34] Bocková M, Slabý J, Špringer T, Homola J (2019). Advances in surface plasmon resonance imaging and microscopy and their biological applications. Annu. Rev. Anal. Chem..

[35] Chrastinová L (2019). A new approach for the diagnosis of myelodysplastic syndrome subtypes based on protein interaction analysis. Sci. Rep..

[36] Chatterjee U, Bose PP, Dey S, Singh TP, Chatterjee BP (2008). Antiproliferative effect of T/Tn specific Artocarpus lakoocha agglutinin (ALA) on human leukemic cells (Jurkat, U937, K562) and their imaging by QD-ALA nanoconjugate. Glycoconj. J..

[37] Roucka M, Zimmermann K, Fido M, Nechansky A (2017). Application of lectin array technology for biobetter characterization: Its correlation with fcγRIII binding and ADCC. Microarrays.

[38] Kuila N (2017). Ecotropic viral integration site I regulates alpha1, 6-fucosyl transferase expression and blocks erythropoiesis in chronic myeloid leukemia. Leuk. Lymphoma.

[39] Patnaik MM, Te A (2017). Refractory anemia with ring sideroblasts ( RARS ) and RARS with thrombocytosis ( RARS-T ): 2017 update on diagnosis, risk-strati fi cation, and management. Am. J. Hematol..

[40] Kizuka Y, Taniguchi N (2016). Enzymes for N-Glycan branching and their genetic and nongenetic regulation in cancer. Biomolecules.

[41] Ihara S (2002). Prometastatic effect of N-acetylglucosaminyltransferase V is due to modification and stabilization of active matriptase by adding β1-6 GlcNAc branching. J. Biol. Chem..

[42] Goldstein IJ, Winter HC, Poretz RD (1997). Chapter 12 plant lectins: Tools for the study of complex carbohydrates. New Compr. Biochem..

[43] Patel PS, Adhvaryu SG, Balar DB, Parikh BJ, Shah PM (1994). Clinical application of serum levels of sialic acid, fucose and seromucoid fraction as tumour markers in human leukemias. Anticancer Res..

[44] Zhou H (2017). Downregulation of miR-224 and let-7i contribute to cell survival and chemoresistance in chronic myeloid leukemia cells by regulating ST3GAL IV expression. Gene.

[45] Barbier V (2020). Endothelial E-selectin inhibition improves acute myeloid leukaemia therapy by disrupting vascular niche-mediated chemoresistance. Nat. Commun..

[46] Ma H (2014). Modification of sialylation is associated with multidrug resistance in human acute myeloid leukemia. Oncogene.

[47] Aller CT, Kucuk O, Springer GF, Gilman-Sachs A (1996). Flow cytometric analysis of T and Tn epitopes on chronic lymphocytic leukemia cells. Am. J. Hematol..

[48] Roy B (2014). On-chip lectin microarray for glycoprofiling of different gastritis types and gastric cancer. Biomicrofluidics.

[49] Simplicien M (2021). The t/tn-specific helix pomatia lectin induces cell death in lymphoma cells negative for t/tn antigens. Cancers.

[50] Piliarik M, Bocková M, Homola J (2010). Surface plasmon resonance biosensor for parallelized detection of protein biomarkers in diluted blood plasma. Biosens. Bioelectron..

[51] Špringer T, Piliarik M, Homola J (2010). Surface plasmon resonance sensor with dispersionless microfluidics for direct detection of nucleic acids at the low femtomole level. Sensors Actuators B Chem..

[52] Pimková K (2012). Surface plasmon resonance biosensor for the detection of VEGFR-1—A protein marker of myelodysplastic syndromes. Anal. Bioanal. Chem..

[53] Herranz S, Bocková M, Marazuela MD, Homola J, Moreno-Bondi MC (2010). An SPR biosensor for the detection of microcystins in drinking water. Anal. Bioanal. Chem..

