# Supplementary material for: Linking aberrant glycosylation of plasma glycoproteins with progression of myelodysplastic syndromes: a study based on plasmonic biosensor and lectin array
Source: Sci Rep. 2023 Aug 7;13:12816. doi: 10.1038/s41598-023-39927-4 (PMC10406930; doi:10.1038/s41598-023-39927-4)
Supplement: Supplementary file 1 — Supplementary Tables. [file 41598_2023_39927_MOESM1_ESM.pdf]

Supplementary Information

Linking aberrant glycosylation of plasma glycoproteins with progression of myelodysplastic syndromes: A study based on plasmonic biosensor and lectin array

Leona Chrastinová[1], Ondřej Pastva[1], Markéta Bocková[2], Hana Kovářová[1], Eliška Ceznerová[1], Roman Kotlín[1], Pavla Pecherková[1], Jana Štikarová[1], Alžběta Hlaváčková[1], Marek Havlíček[1], Jan Válka[1], Jiří Homola[2], Jiří Suttnar[1]*

Supplementary Table S1. *The mean values of SPR responses to all lectins (AAL, ECL, LCH, MAL, VVL, BSL, WGA, UEA, PNA, and HPA) and 90% confidence interval for all tested groups (healthy control, MDS-SLD, MDS-MLD, MDS-EB, AML). CI is placed in parentheses, [mRIU].*

Supplementary Table S2. *The correlations among SPR responses of healthy control plasma to different lectins (AAL, ECL, LCH, MAL, VVL, BSL, WGA, UEA, PNA, and HPA).*

Supplementary Table S3. *The correlations among SPR responses of MDS plasma (MDS-SLD/MLD) to different lectins (AAL, ECL, LCH, MAL, VVL, BSL, WGA, UEA, PNA, and HPA).*

Supplementary Table S4. *The correlations among SPR responses of MDS plasma (MDS-EB) to different lectins (AAL, ECL, LCH, MAL, VVL, BSL, WGA, UEA, PNA, and HPA).*

Supplementary Table S5. *The correlations among SPR responses of AML plasma to different lectins (AAL, ECL, LCH, MAL, VVL, BSL, WGA, UEA, PNA, and HPA).*

Supplementary Table S1.

DG/Lectin	ECL	WGA	LCH	AAL	UEA	MAL	PNA	BSL	VVL	HPA
control	0.09 (0.06,0.11)	0.29 (0.23,0.35)	0.17 (0.14,0.20)	0.23 (0.18,0.28)	0.27 (0.24,0.30)	0.20 (0.13,0.283)	0.04 (0.02,0.05)	0.14 (0.10,0.18)	0.15 (0.11,0.20)	0.36 (0.21,0.52)
MDS-SLD	0.20 (0.15,0.25)	0.33 (0.24,0.41)	0.23 (0.20,0.26)	0.48 (0.41,0.56)	0.24 (0.15,0.33)	0.16 (0.07,0.26)	0.15 (0.01,0.29)	0.19 (0.09,0.29)	0.14 (0.10,0.17)	0.28 (0.14,0.43)
MDS-MLD	0.18 (0.14,0.21)	0.35 (0.30,0.39)	0.22 (0.18,0.27)	0.40 (0.34,0.46)	0.24 (0.20,0.29)	0.20 (0.14,0.26)	0.09 (0.05,0.13)	0.14 (0.10,0.17)	0.16 (0.13,0.19)	0.29 (0.20,0.37)
MDS-EB	0.18 (0.13,0.22)	0.34 (0.31,0.38)	0.22 (0.18,0.25)	0.37 (0.32,0.42)	0.29 (0.25,0.34)	0.30 (0.17,0.44)	0.10 (0.06,0.13)	0.20 (0.12,0.28)	0.17 (0.13,0.22)	0.27 (0.19,0.35)
AML	0.17 (0.13,0.22)	0.33 (0.29,0.38)	0.21 (0.19,0.22)	0.39 (0.34,0.44)	0.37 (0.23,0.51)	0.33 (0.15,0.52)	0.10 (0.07,0.12)	0.27 (0.12,0.42)	0.24 (0.12,0.36)	0.24 (0.16,0.33)

Supplementary Table S2.

	AAL	ECL	LCH	MAL	VVL	BSL	WGA	UEA	PNA	HPA
AAL	1	0.549602156	0.737829311	0.22385343	0.505569419	-0.106546092	0.511676335	0.464040271	0.052528909	0.156081244
ECL	0.549602156	1	0.666327087	0.041674427	-0.048607485	-0.286009535	0.108095326	0.328544246	0.224613113	0.342727174
LCH	0.737829311	0.666327087	1	0.195558826	0.215497772	-0.186049812	0.097094009	0.551186714	0.491021258	0.410865066
MAL	0.22385343	0.041674427	0.195558826	1	0.56903435	0.47686186	-0.085733529	0.496020627	-0.206742995	0.171947542
VVL	0.505569419	-0.048607485	0.215497772	0.56903435	1	0.109434456	0.626425835	0.053201042	-0.271870212	-0.022951473
BSL	-0.106546092	-0.286009535	-0.186049812	0.47686186	0.109434456	1	-0.267566983	0.494796295	-0.165840471	-0.072487937
WGA	0.511676335	0.108095326	0.097094009	-0.085733529	0.626425835	-0.267566983	1	-0.288121152	-0.286901254	0.051509007
UEA	0.464040271	0.328544246	0.551186714	0.496020627	0.053201042	0.494796295	-0.288121152	1	0.060696406	0.050130667
PNA	0.052528909	0.224613113	0.491021258	-0.206742995	-0.271870212	-0.165840471	-0.286901254	0.060696406	1	0.328170228
HPA	0.156081244	0.342727174	0.410865066	0.171947542	-0.022951473	-0.072487937	0.051509007	0.050130667	0.328170228	1

Supplementary Table S3.

	AAL	ECL	LCH	MAL	VVL	BSL	WGA	UEA	PNA	HPA
AAL	1	0.53534177	0.69767557	0.160099852	0.527756789	-0.120203201	-0.045247431	-0.271132937	0.093683699	-0.120401788
ECL	0.53534177	1	0.824929719	0.638733771	0.467137686	0.067609051	-0.011728153	-0.021120698	0.130856388	-0.25160526
LCH	0.69767557	0.824929719	1	0.420368845	0.565076921	-0.005223692	-0.007662952	-0.0894806	0.181156932	-0.266094543
MAL	0.160099852	0.638733771	0.420368845	1	-0.001214461	-0.221971421	-0.099752792	-0.169869296	-0.230062673	0.006063453
VVL	0.527756789	0.467137686	0.565076921	-0.001214461	1	-0.030062519	0.077173998	-0.054343761	0.333537066	0.06730403
BSL	-0.120203201	0.067609051	-0.005223692	-0.221971421	-0.030062519	1	0.135295722	0.609196034	0.405519217	-0.334154762
WGA	-0.045247431	-0.011728153	-0.007662952	-0.099752792	0.077173998	0.135295722	1	0.118655926	0.234851562	0.169235828
UEA	-0.271132937	-0.021120698	-0.0894806	-0.169869296	-0.054343761	0.609196034	0.118655926	1	0.493399703	-0.366184512
PNA	0.093683699	0.130856388	0.181156932	-0.230062673	0.333537066	0.405519217	0.234851562	0.493399703	1	-0.244656883
HPA	-0.120401788	-0.25160526	-0.266094543	0.006063453	0.06730403	-0.334154762	0.169235828	-0.366184512	-0.244656883	1

Supplementary Table S4.

	AAL	ECL	LCH	MAL	VVL	BSL	WGA	UEA	PNA	HPA
AAL	1	0.526956366	0.5714756	0.534766612	0.554037089	0.513415412	0.048822025	0.406252615	-0.132706511	-0.364041125
ECL	0.526956366	1	0.706469778	0.579656755	0.665677935	0.199733084	0.115756504	0.496455571	0.211997847	-0.186021931
LCH	0.5714756	0.706469778	1	0.45118921	0.518725902	0.499707559	-0.039196301	0.35470935	0.027094208	-0.030136789
MAL	0.534766612	0.579656755	0.45118921	1	0.771575512	0.457418655	-0.007831549	0.666563633	-0.155270424	-0.442016555
VVL	0.554037089	0.665677935	0.518725902	0.771575512	1	0.679086429	-0.029680695	0.843900245	0.004216566	-0.293961997
BSL	0.513415412	0.199733084	0.499707559	0.457418655	0.679086429	1	-0.130884756	0.586031427	-0.001034764	-0.118704042
WGA	0.048822025	0.115756504	-0.039196301	-0.007831549	-0.029680695	-0.130884756	1	-0.01238677	0.161946761	-0.019356955
UEA	0.406252615	0.496455571	0.35470935	0.666563633	0.843900245	0.586031427	-0.01238677	1	0.02089077	-0.051345101
PNA	-0.132706511	0.211997847	0.027094208	-0.155270424	0.004216566	-0.001034764	0.161946761	0.02089077	1	0.273333189
HPA	-0.364041125	-0.186021931	-0.030136789	-0.442016555	-0.293961997	-0.118704042	-0.019356955	-0.051345101	0.273333189	1

Supplementary Table S5.

	AAL	ECL	LCH	MAL	VVL	BSL	WGA	UEA	PNA	HPA
AAL	1	0.115007493	0.54133207	-0.043542245	0.061603307	-0.068765921	-0.155396861	-0.115985137	-0.024248068	-0.204589191
ECL	0.115007493	1	0.770619323	0.72805128	0.821209463	0.829614815	0.445347614	0.77424103	0.296175052	-0.001968048
LCH	0.54133207	0.770619323	1	0.553723024	0.576222225	0.568955334	0.373012939	0.516291535	0.298996667	0.058591523
MAL	-0.043542245	0.72805128	0.553723024	1	0.916356423	0.930046674	0.326352996	0.951433452	0.094894512	0.1598263
VVL	0.061603307	0.821209463	0.576222225	0.916356423	1	0.918611801	0.262960713	0.944662008	0.127702852	-0.06587815
BSL	-0.068765921	0.829614815	0.568955334	0.930046674	0.918611801	1	0.487068285	0.949688688	0.204373334	0.080447171
WGA	-0.155396861	0.445347614	0.373012939	0.326352996	0.262960713	0.487068285	1	0.392916043	0.229411291	0.228238028
UEA	-0.115985137	0.77424103	0.516291535	0.951433452	0.944662008	0.949688688	0.392916043	1	0.185918981	0.126936719
PNA	-0.024248068	0.296175052	0.298996667	0.094894512	0.127702852	0.204373334	0.229411291	0.185918981	1	0.275558368
HPA	-0.204589191	-0.001968048	0.058591523	0.1598263	-0.06587815	0.080447171	0.228238028	0.126936719	0.275558368	1
